# Crystal structure and Hirshfeld surface analysis of 3-(3-hy­droxy­phen­yl)-1-(1*H*-pyrrol-2-yl)prop-2-en-1-one hemihydrate

**DOI:** 10.1107/S2056989023001925

**Published:** 2023-03-10

**Authors:** Ahmad Zaidi Ismail, Mohanapriya Gunasekharan, Thiruventhan Karunakaran, Siti Munirah Mohd Faudzi

**Affiliations:** a School of Chemical Sciences, University of Auckland, Private Bag 92019, Auckland, New Zealand; b Department of Chemistry, Faculty of Science, Universiti Putra Malaysia, Serdang, 43400, Selangor, Malaysia; c Centre for Drug Research, Universiti Sains Malaysia, 11800 USM, Pulau Pinang, Malaysia; d Natural Medicines and Product Research Laboratory, Institute of Bioscience, Universiti Putra Malaysia, Serdang, 43400, Selangor, Malaysia; Universität Greifswald, Germany

**Keywords:** chalcone, pyrrole-derived chalcone, pyrrole, crystal structure, SCXRD

## Abstract

High-quality single crystals of the title compound, 2C_13_H_11_NO_2_·H_2_O, were grown and a structural analysis was performed. The asymmetric unit comprises one mol­ecule of 3-(3-hy­droxy­phen­yl)-1-(1*H*-pyrrol-2-yl)prop-2-en-1-one (**3HPPP**), which was recently discovered to be a promising anti-MRSA candidate, and a half-mol­ecule of water. The compound crystallizes in the monoclinic space group *P*2/*c*. The crystal structure features inter­molecular pyrrole-N—H⋯O (water), carbon­yl/keto-C—O⋯H—O-phenol and phenol-C—O⋯H (water) hydrogen bonds, which help to consolidate the crystal packing. A Hirshfeld surface analysis for the components in the asymmetric unit showed that H⋯H (40.9%) and H⋯C/C⋯H (32.4%) contacts make the largest contributions to the inter­molecular inter­actions of **3HPPP**. Considering the presence of water, in its vicinity H⋯O/O⋯H and H⋯C/C⋯H are the most significant contacts, contributing 48.7 and 29.8%, respectively.

## Abstract 2

3-(3-Hy­droxy­phen­yl)-1-(1*H*-pyrrol-2-yl)prop-2-en-1-one (**3HPPP**) crystallized as planar mol­ecule together with half a mol­ecule of water in the asymmetric unit in the monoclinic crystal system with space group *P*2/*c*. A Hirshfeld surface analysis for the chalcone component showed that H⋯H (40.9%) and H⋯C/C⋯H (32.4%) contacts make the largest contributions to the crystal packing of **3HPPP**. In the vicinity of water, the H⋯O/O⋯H and H⋯C/C⋯H contacts are the most significant, at 48.7% and 29.8%, respectively.

## 1. Chemical context

Chalcones are 1,3-diphenyl-2-propen-1-ones with an α,β-unsaturated carbonyl system in between two aromatic rings (Zhuang *et al.*, 2017 ▸; Attarde *et al.*, 2010 ▸). Chalcones are widely used as precursors for the biosynthesis of compounds in the flavonoid class, and can be chemically synthesized by various reactions such as aldol condensation, and Suzuki and Wittig reactions (Zhuang *et al.*, 2017 ▸). To date, chalcones have continued to attract great inter­est from researchers because of their simple chemistry and diverse applications in medicinal and synthetic chemistry (Zhuang *et al.*, 2017 ▸), analytical chemistry (Sun *et al.*, 2012 ▸), materials chemistry and lighting technology (Anandkumar *et al.*, 2017 ▸; Danko *et al.*, 2012 ▸). 
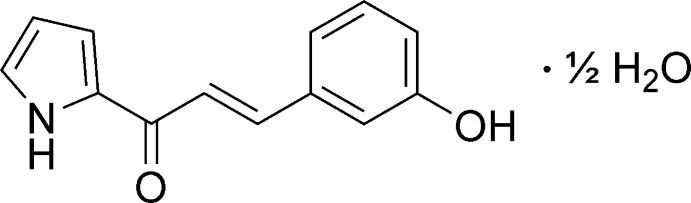


Chalcone analogues have been reported with a wide range of biological activities, including anti-inflammatory, anti­microbial, and anti­cancer properties (Kar Mahapatra *et al.*, 2019 ▸; Lin *et al.*, 2002 ▸; Nowakowska, 2007 ▸). Recently, we discovered a new promising anti-microbial candidate, 3-(3-hy­droxy­phen­yl)-1-(1*H*-pyrrol-2-yl)prop-2-en-1-one (**3HPPP**), which showed remarkable inhibitory activity on methicillin-resistant *Staphylococcus aureus* (MRSA, ATCC 700699) with MIC and MBC values of 0.23 mg ml^−1^ and 0.47 mg ml^−1^, respectively (Gunasekharan *et al.*, 2021 ▸). However, as yet the crystal structure of this compound has remained elusive. The mol­ecular structure of its hydrate is analysed and discussed herein.

## 2. Structural commentary

**Figure 1:**
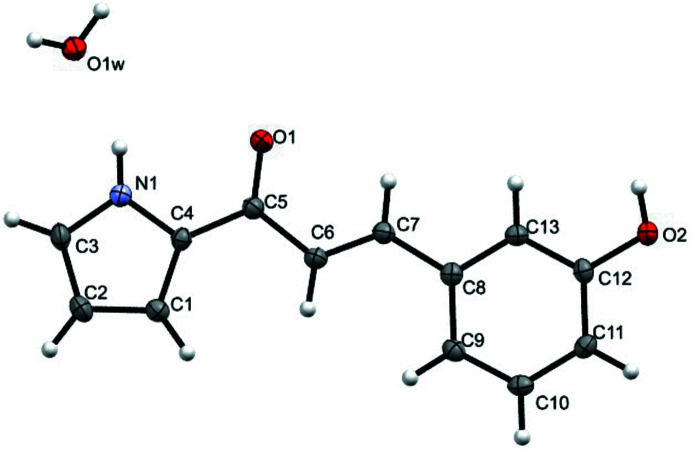
*ORTEP* (Burnett & Johnson, 1996 ▸) diagram of compound **3HPPP** plus the symmetry-completed water molecule with the atom-labelling scheme and 50% probability ellipsoids.

The mol­ecular structure of the asymmetric unit of **3HPPP** plus the symmetry-completed water mol­ecule are shown in Fig. 1 ▸. The asymmetric unit consists of a mol­ecule of **3HPPP** in a neutral state plus half a water mol­ecule of crystallization. The investigated bioactive compound crystallized in the monoclinic crystal system, space group *P*2/*c*, with the unit cell containing four mol­ecules of **3HPPP** together with two mol­ecules of water. Four water mol­ecules reside on four of the cell edges on the crystallographic *c*-axis and are shared between the unit and adjacent cells. Further analysis of the metrical parameters of the mol­ecule showed no anomalies compared to the available literature data for related compounds. The planarity of **3HPPP** is confirmed as both the aromatic pyrrole (N1/C1–C4) and phenyl (C8–C13) rings are aligned in the plane of the aliphatic α,β-unsaturated ketone linker, making dihedral angles of 0.91 (7) and 5.98 (7)°, respectively with the linker.

## 3. Supra­molecular features

**Figure 2:**
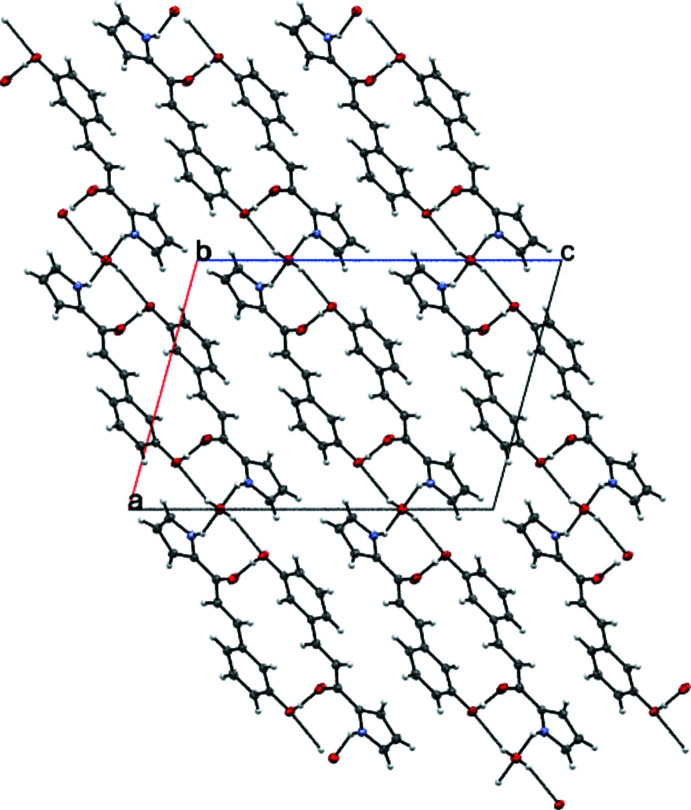
The crystal packing of compound **3HPPP** viewed along the *b* axis. The inter­molecular inter­actions are indicated by dashed lines.

Fig. 2 ▸ illustrates the unit cell of **3HPPP** viewed along the crystallographic *b*-axis and the supra­molecular association within and around it. In the crystal, mol­ecules are linked into dimers *via* multiple inter­molecular hydrogen bonds (Table 1 ▸). The dimers are arranged in planes with two distinct orientations and at an angle of roughly 61° to each other, while the water mol­ecules act as hinges. This represents a zigzag pattern when viewed along the the *ac* diagonal. Furthermore, the **3HPPP** dimers are arranged in a stair-like fashion, which ascends/descends roughly in the *b*-axis direction. Inter­molecular hydrogen bonds C13—H13⋯O1^i^ [symmetry code: (i) −*x* + 1, −*y* + 1, −*z* + 1) between two mol­ecules of **3HPPP** can be observed connecting these non-covalently. Mol­ecules of **3HPPP** are linked into inversion dimer–dimer chains through these weak inter­actions. Moreover, the lattice water mol­ecules act as donors and acceptors in hydrogen bonds with the phenol and pyrrole moieties of **3HPPP** [O3—H3*O*⋯O2^ii^ and N1—H1*N*⋯O3; symmetry code: (ii) *x* − 1, −*y* + 1, *z* − 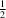 ; Table 2 ▸]. All hydrogen atoms and all lone pairs of the water mol­ecule are engaged in hydrogen bonding (Fig. 2 ▸). These hydrogen bonds connect two of the **3HPPP** dimers in different planes comparably strongly and further consolidate the crystal packing.

## 4. Database survey

A database survey of the Cambridge Structural Database (WEBCSD version 1.9.32, updated September 2022; Groom *et al.*, 2016 ▸) revealed that no structure of a compound with a close similarity to the entire **3HPPP** mol­ecule as been reported. However, focusing on the pyrrole ene-one side yielded three [refcodes HIXGAW (Norsten *et al.*, 1999 ▸); RICFEP (Camarillo *et al.*, 2007 ▸) and RICFEP01 (Jones, 2013 ▸)] similar compounds with a 77–88% similarity score relative to the title compound. The title compound differs from those at the substituted ethyl-phenol (C6–C13) side. The overall conformation of the title compound and HIXGAW are very nearly planar and the other two (RICFEP and RICFEP01) are planar. A notable difference relates to the substitution on the keto side. The respective dihedral angles in the studied compound and in HIXGAW are in the range of 5.49–24.65°.

## 5. Hirshfeld surface analysis

**Figure 3:**
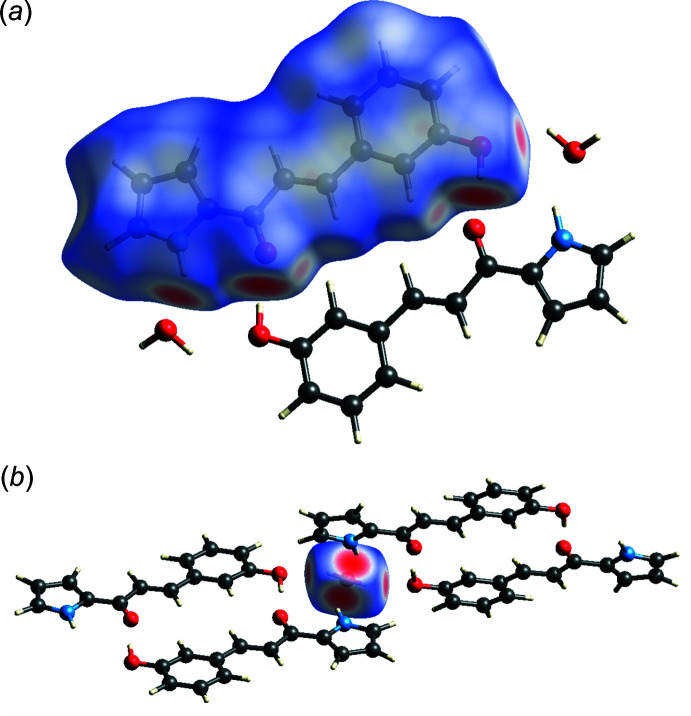
Three-dimensional Hirshfeld surfaces plotted over *d*_norm_ in the range −0.667 to 1.118 a.u of (*a*) compound **3HPPP** and (*b*) the water mol­ecule, generated with *Crystal Explorer* (Spackman *et al.*, 2021 ▸).

*Crystal Explorer 21* (Spackman *et al.*, 2021 ▸) was used to calculate the Hirshfeld surfaces to obtain further insight into the inter­molecular inter­actions in the crystal structure of the title compound. The three-dimensional Hirshfeld surfaces plotted over *d*_norm_ ranging from −0.667 to 1.118 a.u. are shown in Fig. 3 ▸. For compound **3HPPP** (Fig. 3 ▸ *a*), the most prominent inter­actions in the crystal packing are the hydrogen bonds, which are represented by four bright-red spots on the mapped *d*_norm_ surface. The bright-red spots around O1 and O2 correspond to the hydrogen bonding between hydroxyl and carbon­yl/keto functional groups of two mol­ecules of **3HPPP**. The other two bright-red spots are due to hydrogen bonding between the pyrrole-N—H functional group and the water mol­ecule, and between the water mol­ecule and the hydroxyl group of **3HPPP**. In addition to these four spots, two faint-red spots appear around O1 and H13, representing the non-classical hydrogen-bond inter­action of an aromatic C–H and the carbon­yl/keto functional group. The intensities of all these red spots indicate the relative strengths of the inter­actions, as well as the distances of the contacts. The *d*_norm_ Hirshfeld surface for the water mol­ecules present in the crystal lattice was also calculated and mapped (Fig. 3 ▸ *b*). Four bright-red spots are observed, which are due to the pyrrole-to-water and water-to-hydroxyl hydrogen bonds and are thereby mirrors of the inter­actions involving water described above.

**Figure 4:**
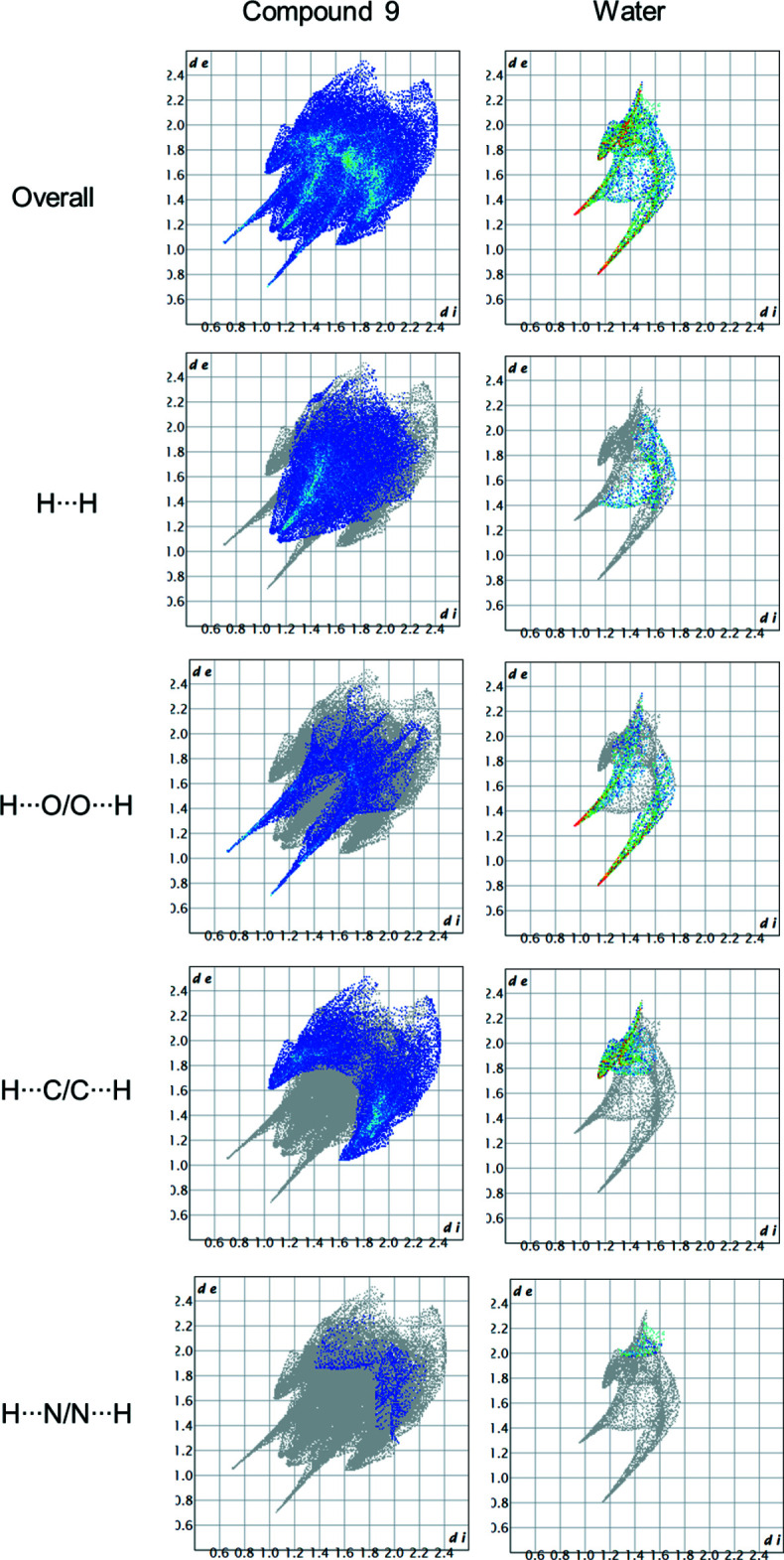
Overall two-dimensional fingerprint plots for compound **3HPPP** and the water mol­ecule together with those delineated into H⋯H, H⋯O/O⋯H, H⋯C/C⋯H and H⋯N/N⋯H inter­actions, generated with *Crystal Explorer* (Spackman *et al.*, 2021 ▸).

The overall two-dimensional fingerprint plots of both mol­ecules, water and **3HPPP**, and those delineated into H⋯H, H⋯O/O⋯H, H⋯C/C⋯H and H⋯N/N⋯H inter­actions are shown in Fig. 4 ▸, while the percentage contributions are listed in Table 2 ▸. The two-dimensional fingerprint plots for compound **3HPPP** show that H⋯H and H⋯ C/C⋯ H are the most significant inter­atomic inter­actions in the crystal packing, contributing 40.9 and 32.4%, respectively, to the Hirshfeld surface. The H⋯O/O⋯H (19.4%) and other minor contacts (H⋯N/N⋯H = 2.0%) further contribute to the Hirshfeld surfaces. On the other hand, the most prominent inter­atomic contacts for the water mol­ecule are H⋯O/O⋯H, as expected, with a 48.7% contribution while H⋯H and H⋯C/C⋯H contacts contribute 16.2 and 29.8%, respectively.

## 6. Synthesis and crystallization

**Figure 5:**
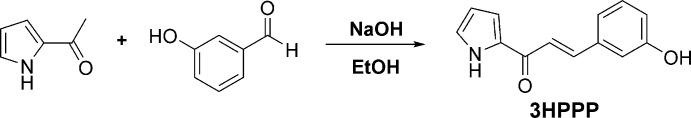
Synthetic route towards 3-(3-hy­droxy­phen­yl)-1-(1*H*-pyrrol-2-yl)prop-2-en-1-one (**3HPPP**).

The 3-hy­droxy­pyrrolylated chalcone **3HPPP** was synthesized by a Claisen–Schmidt condensation reaction between 2-ace­tyl­pyrrole (2 mmol) and 3-hy­droxy­benzaldehyde (2 mmol) under ethano­lic (10 ml) conditions. The resulting mixture was stirred for 5 min followed by the dropwise addition of 3 ml of a 40% aqueous NaOH solution (Fig. 5 ▸). The mixture was stirred overnight at room temperature. After the reaction was essentially complete, it was quenched by pouring the resultant solution onto crushed ice and extraction with ethyl acetate (3 × 10 ml). The organic layer was washed with distilled water (3 × 10 ml), filtered, dried over anhydrous MgSO_4_ and concentrated *in vacuo*. Finally, the collected crudes were purified by gravity column chromatography using hexa­ne:ethyl acetate (ratio of 7:3) as solvent system. Multiple spectroscopic analyses confirmed the chemical structure (Mohd Faudzi *et al.*, 2020 ▸). The obtained pure **3HPPP** was then recrystallized by slow evaporation of an ethanol solution, giving crystals suitable for X-ray diffraction analysis.

## 7. Refinement

Crystal data, data collection and structure refinement details are summarized in Table 3 ▸. The hydrogen atoms bound to oxygen or nitro­gen were found in difference maps and refined freely. The carbon-bound hydrogen atoms, which are all aromatic, were geometrically placed and refined using a riding model with C—H = 0.95 Å and *U*_iso_(H) = 1.2*U*_eq_(C).

## Supplementary Material

Crystal structure: contains datablock(s) I. DOI: 10.1107/S2056989023001925/yz2023sup1.cif

Structure factors: contains datablock(s) I. DOI: 10.1107/S2056989023001925/yz2023Isup2.hkl

CCDC reference: 2233979

Additional supporting information:  crystallographic information; 3D view; checkCIF report

## Figures and Tables

**Table 1:** Hydrogen-bond geometry (Å, °)

*D*—H⋯*A*	*D*—H	H⋯*A*	*D*⋯*A*	*D*—H⋯*A*
O2—H2*O*⋯O1^i^	0.901 (19)	1.828 (19)	2.7257 (11)	174.2 (16)
N1—H1*N*⋯O3	0.888 (17)	2.041 (17)	2.8722 (13)	155.4 (14)
C13—H13⋯O1^i^	0.95	2.51	3.2050 (13)	130
O3—H3*O*⋯O2^ii^	0.864 (18)	2.320 (18)	2.9430 (7)	129.2 (16)

Symmetry codes: (i) 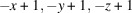 ; (ii) 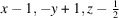 .

**Table 2:** Percentage contribution of inter­atomic contacts to the calculated Hirshfeld surfaces for the individual constituents in the asymmetric unit of **3HPPP**

Contact	Percentage contribution	
	**3HPPP**	Water
H⋯H	40.9	16.2
H⋯O/O⋯H	19.4	48.7
H⋯C/C⋯H	32.4	29.8
H⋯N/N⋯H	2.0	4.6

**Table 3:** Experimental details

Crystal data	
Chemical formula	2C_13_H_11_NO_2_·H_2_O
*M*_r_	444.47
Crystal system, space group	Monoclinic, *P*2/*c*
Temperature (K)	100
*a*, *b*, *c* (Å)	11.9096 (1), 5.5836 (1), 16.8121 (2)
β (°)	105.356 (1)
*V* (Å^3^)	1078.07 (3)
*Z*	2
Radiation type	Cu *K*α
μ (mm^−1^)	0.78
Crystal size (mm)	0.18 × 0.17 × 0.13

Data collection	
Diffractometer	XtaLAB Synergy, Dualflex, AtlasS2
Absorption correction	Gaussian (*CrysAlis PRO*; Rigaku OD, 2021 ▸)
*T*_min_, *T*_max_	0.676, 1.000
No. of measured, independent and observed [*I* > 2σ(*I*)] reflections	13888, 2221, 2067
*R*_int_	0.028
(sin θ/λ)_max_ (Å^−1^)	0.627

Refinement	
*R*[*F* ^2^ > 2σ(*F* ^2^)], *wR*(*F* ^2^), *S*	0.034, 0.095, 1.04
No. of reflections	2221
No. of parameters	163
H-atom treatment	H atoms treated by a mixture of independent and constrained refinement
Δρ_max_, Δρ_min_ (e Å^−3^)	0.30, −0.19

Computer programs: *CrysAlis PRO* (Rigaku OD, 2021 ▸), *SHELXT2018/2* (Sheldrick, 2015*a* ▸), *SHELXL2018/3* (Sheldrick, 2015*b* ▸), *OLEX2* 1.3 (Dolomanov *et al.*, 2009 ▸) and *publCIF* (Westrip, 2010 ▸).

## supplementary crystallographic information

### Crystal data

**Table:**

2C_13_H_11_NO_2_·H_2_O	*F*(000) = 468
*M**_r_* = 444.47	*D*_x_ = 1.369 Mg m^−^^3^
Monoclinic, *P*2/*c*	Cu *K*α radiation, λ = 1.54184 Å
*a* = 11.9096 (1) Å	Cell parameters from 9119 reflections
*b* = 5.5836 (1) Å	θ = 3.8–76.1°
*c* = 16.8121 (2) Å	µ = 0.78 mm^−^^1^
β = 105.356 (1)°	*T* = 100 K
*V* = 1078.07 (3) Å^3^	Prism, colourless
*Z* = 2	0.18 × 0.17 × 0.13 mm

### Data collection

**Table:**

XtaLAB Synergy, Dualflex, AtlasS2 diffractometer	2067 reflections with *I* > 2σ(*I*)
Radiation source: micro-focus sealed X-ray tube	*R*_int_ = 0.028
ω scans	θ_max_ = 75.2°, θ_min_ = 3.9°
Absorption correction: gaussian (CrysAlisPro; Rigaku OD, 2021)	*h* = −14→14
*T*_min_ = 0.676, *T*_max_ = 1.000	*k* = −6→7
13888 measured reflections	*l* = −18→21
2221 independent reflections	

### Refinement

**Table:**

Refinement on *F*^2^	Secondary atom site location: difference Fourier map
Least-squares matrix: full	Hydrogen site location: mixed
*R*[*F*^2^ > 2σ(*F*^2^)] = 0.034	H atoms treated by a mixture of independent and constrained refinement
*wR*(*F*^2^) = 0.095	*w* = 1/[σ^2^(*F*_o_^2^) + (0.0512*P*)^2^ + 0.3933*P*] where *P* = (*F*_o_^2^ + 2*F*_c_^2^)/3
*S* = 1.04	(Δ/σ)_max_ < 0.001
2221 reflections	Δρ_max_ = 0.30 e Å^−^^3^
163 parameters	Δρ_min_ = −0.19 e Å^−^^3^
0 restraints	Extinction correction: *SHELXL2018/3* (Sheldrick, 2015b), Fc^*^=kFc[1+0.001xFc^2^λ^3^/sin(2θ)]^-1/4^
Primary atom site location: dual	Extinction coefficient: 0.0039 (5)

### Special details

**Table:**

Geometry. All esds (except the esd in the dihedral angle between two l.s. planes) are estimated using the full covariance matrix. The cell esds are taken into account individually in the estimation of esds in distances, angles and torsion angles; correlations between esds in cell parameters are only used when they are defined by crystal symmetry. An approximate (isotropic) treatment of cell esds is used for estimating esds involving l.s. planes.

### Fractional atomic coordinates and isotropic or equivalent isotropic displacement parameters (Å^2^)

**Table:**

	*x*	*y*	*z*	*U*_iso_*/*U*_eq_	
O1	0.27900 (7)	0.42189 (15)	0.33951 (5)	0.0268 (2)	
O2	0.81284 (7)	0.95369 (15)	0.59533 (5)	0.0215 (2)	
O3	0.000000	0.0526 (2)	0.250000	0.0208 (3)	
N1	0.10566 (8)	0.45548 (17)	0.19188 (5)	0.0186 (2)	
C1	0.17738 (9)	0.7925 (2)	0.15408 (6)	0.0178 (2)	
H1A	0.225141	0.928386	0.153517	0.021*	
C2	0.07695 (9)	0.7278 (2)	0.09255 (6)	0.0222 (3)	
H2A	0.044033	0.812012	0.042726	0.027*	
C3	0.03512 (9)	0.5190 (2)	0.11809 (6)	0.0222 (3)	
H3	−0.032172	0.434067	0.088590	0.027*	
C4	0.19382 (9)	0.62052 (19)	0.21594 (6)	0.0159 (2)	
C5	0.28203 (9)	0.59293 (19)	0.29291 (6)	0.0177 (2)	
C6	0.37555 (9)	0.7736 (2)	0.31381 (6)	0.0179 (2)	
H6	0.376212	0.901776	0.276763	0.021*	
C7	0.45937 (9)	0.75864 (19)	0.38443 (6)	0.0173 (2)	
H7	0.455306	0.624717	0.418413	0.021*	
C8	0.55672 (9)	0.92326 (19)	0.41588 (6)	0.0155 (2)	
C9	0.56928 (9)	1.14020 (19)	0.37719 (6)	0.0174 (2)	
H9	0.513693	1.186081	0.327786	0.021*	
C10	0.66350 (9)	1.28752 (19)	0.41156 (6)	0.0184 (2)	
H10	0.672229	1.433834	0.384975	0.022*	
C11	0.74552 (9)	1.22472 (19)	0.48433 (6)	0.0181 (2)	
H11	0.809769	1.326921	0.507080	0.022*	
C12	0.73238 (9)	1.0110 (2)	0.52331 (6)	0.0166 (2)	
C13	0.63891 (9)	0.86102 (19)	0.48899 (6)	0.0161 (2)	
H13	0.630823	0.714363	0.515581	0.019*	
H2O	0.7856 (15)	0.832 (3)	0.6202 (11)	0.050 (5)*	
H1N	0.0948 (13)	0.324 (3)	0.2186 (9)	0.034 (4)*	
H3O	−0.0335 (16)	−0.039 (4)	0.2091 (11)	0.062 (6)*	

### Atomic displacement parameters (Å^2^)

**Table:**

	*U*^11^	*U*^22^	*U*^33^	*U*^12^	*U*^13^	*U*^23^
O1	0.0251 (4)	0.0272 (5)	0.0228 (4)	−0.0081 (3)	−0.0032 (3)	0.0089 (3)
O2	0.0178 (4)	0.0239 (4)	0.0194 (4)	−0.0044 (3)	−0.0012 (3)	0.0032 (3)
O3	0.0218 (5)	0.0193 (6)	0.0208 (5)	0.000	0.0046 (4)	0.000
N1	0.0170 (4)	0.0199 (5)	0.0176 (4)	−0.0025 (4)	0.0025 (3)	0.0010 (4)
C1	0.0176 (5)	0.0187 (5)	0.0171 (5)	0.0015 (4)	0.0044 (4)	0.0011 (4)
C2	0.0214 (5)	0.0256 (6)	0.0167 (5)	0.0024 (4)	0.0000 (4)	0.0025 (4)
C3	0.0172 (5)	0.0277 (6)	0.0182 (5)	−0.0011 (4)	−0.0015 (4)	−0.0008 (4)
C4	0.0145 (5)	0.0173 (5)	0.0163 (5)	−0.0006 (4)	0.0045 (4)	−0.0010 (4)
C5	0.0173 (5)	0.0189 (5)	0.0167 (5)	0.0002 (4)	0.0043 (4)	0.0013 (4)
C6	0.0183 (5)	0.0185 (5)	0.0166 (5)	−0.0009 (4)	0.0040 (4)	0.0019 (4)
C7	0.0176 (5)	0.0170 (5)	0.0176 (5)	0.0003 (4)	0.0051 (4)	0.0014 (4)
C8	0.0153 (5)	0.0165 (5)	0.0156 (5)	0.0011 (4)	0.0056 (4)	−0.0016 (4)
C9	0.0190 (5)	0.0180 (5)	0.0150 (5)	0.0017 (4)	0.0043 (4)	0.0002 (4)
C10	0.0232 (5)	0.0144 (5)	0.0191 (5)	−0.0002 (4)	0.0083 (4)	0.0007 (4)
C11	0.0176 (5)	0.0176 (5)	0.0195 (5)	−0.0038 (4)	0.0055 (4)	−0.0030 (4)
C12	0.0150 (5)	0.0196 (5)	0.0147 (5)	0.0017 (4)	0.0034 (4)	−0.0014 (4)
C13	0.0172 (5)	0.0147 (5)	0.0170 (5)	0.0005 (4)	0.0059 (4)	0.0008 (4)

### Geometric parameters (Å, º)

**Table:**

O1—C5	1.2417 (13)	C5—C6	1.4748 (14)
O2—C12	1.3683 (12)	C6—C7	1.3362 (14)
O2—H2O	0.901 (19)	C6—H6	0.9500
O3—H3O	0.864 (18)	C7—C8	1.4642 (14)
O3—H3O^i^	0.864 (18)	C7—H7	0.9500
N1—C3	1.3484 (13)	C8—C13	1.3973 (14)
N1—C4	1.3749 (13)	C8—C9	1.4015 (15)
N1—H1N	0.888 (17)	C9—C10	1.3879 (15)
C1—C4	1.3906 (14)	C9—H9	0.9500
C1—C2	1.4056 (14)	C10—C11	1.3932 (14)
C1—H1A	0.9500	C10—H10	0.9500
C2—C3	1.3804 (17)	C11—C12	1.3902 (15)
C2—H2A	0.9500	C11—H11	0.9500
C3—H3	0.9500	C12—C13	1.3903 (15)
C4—C5	1.4429 (14)	C13—H13	0.9500
			
C12—O2—H2O	109.4 (11)	C5—C6—H6	119.7
H3O—O3—H3O^i^	108 (3)	C6—C7—C8	127.97 (10)
C3—N1—C4	109.61 (9)	C6—C7—H7	116.0
C3—N1—H1N	122.9 (10)	C8—C7—H7	116.0
C4—N1—H1N	127.4 (10)	C13—C8—C9	119.15 (9)
C4—C1—C2	107.27 (10)	C13—C8—C7	117.69 (9)
C4—C1—H1A	126.4	C9—C8—C7	123.14 (9)
C2—C1—H1A	126.4	C10—C9—C8	119.53 (9)
C3—C2—C1	107.15 (9)	C10—C9—H9	120.2
C3—C2—H2A	126.4	C8—C9—H9	120.2
C1—C2—H2A	126.4	C9—C10—C11	121.19 (10)
N1—C3—C2	108.67 (10)	C9—C10—H10	119.4
N1—C3—H3	125.7	C11—C10—H10	119.4
C2—C3—H3	125.7	C12—C11—C10	119.33 (10)
N1—C4—C1	107.30 (9)	C12—C11—H11	120.3
N1—C4—C5	120.68 (9)	C10—C11—H11	120.3
C1—C4—C5	132.01 (10)	O2—C12—C11	118.55 (9)
O1—C5—C4	120.80 (10)	O2—C12—C13	121.51 (10)
O1—C5—C6	121.48 (9)	C11—C12—C13	119.94 (9)
C4—C5—C6	117.73 (9)	C12—C13—C8	120.84 (10)
C7—C6—C5	120.54 (10)	C12—C13—H13	119.6
C7—C6—H6	119.7	C8—C13—H13	119.6
			
C4—C1—C2—C3	−0.20 (12)	C5—C6—C7—C8	−178.53 (9)
C4—N1—C3—C2	0.25 (13)	C6—C7—C8—C13	−175.38 (10)
C1—C2—C3—N1	−0.03 (13)	C6—C7—C8—C9	6.06 (17)
C3—N1—C4—C1	−0.38 (12)	C13—C8—C9—C10	0.72 (14)
C3—N1—C4—C5	−179.58 (9)	C7—C8—C9—C10	179.26 (9)
C2—C1—C4—N1	0.35 (12)	C8—C9—C10—C11	−0.54 (15)
C2—C1—C4—C5	179.42 (11)	C9—C10—C11—C12	−0.24 (16)
N1—C4—C5—O1	−1.17 (16)	C10—C11—C12—O2	−179.10 (9)
C1—C4—C5—O1	179.86 (11)	C10—C11—C12—C13	0.84 (15)
N1—C4—C5—C6	178.55 (9)	O2—C12—C13—C8	179.27 (9)
C1—C4—C5—C6	−0.42 (17)	C11—C12—C13—C8	−0.67 (15)
O1—C5—C6—C7	−0.66 (16)	C9—C8—C13—C12	−0.12 (15)
C4—C5—C6—C7	179.62 (10)	C7—C8—C13—C12	−178.74 (9)

Symmetry code: (i) −*x*, *y*, −*z*+1/2.

### Hydrogen-bond geometry (Å, º)

**Table:**

*D*—H···*A*	*D*—H	H···*A*	*D*···*A*	*D*—H···*A*
O2—H2*O*···O1^ii^	0.901 (19)	1.828 (19)	2.7257 (11)	174.2 (16)
N1—H1*N*···O3	0.888 (17)	2.041 (17)	2.8722 (13)	155.4 (14)
C13—H13···O1^ii^	0.95	2.51	3.2050 (13)	130
O3—H3*O*···O2^iii^	0.864 (18)	2.320 (18)	2.9430 (7)	129.2 (16)

Symmetry codes: (ii) −*x*+1, −*y*+1, −*z*+1; (iii) *x*−1, −*y*+1, *z*−1/2.
